# ARNT Inhibits H5N1 Influenza A Virus Replication by Interacting with the PA Protein

**DOI:** 10.3390/v14071347

**Published:** 2022-06-21

**Authors:** Huapeng Feng, Zeng Wang, Pengyang Zhu, Li Wu, Jianzhong Shi, Yanbing Li, Jianhong Shu, Yulong He, Huihui Kong

**Affiliations:** 1 Department of Biopharmacy, College of Life Sciences and Medicine, Zhejiang Sci-Tech University, Hangzhou 310018, China; fenghuapeng@zstu.edu.cn (H.F.); shujh1978@163.com (J.S.); heyulong2003@163.com (Y.H.); 2 State Key Laboratory of Veterinary Biotechnology, Harbin Veterinary Research Institute, Chinese Academy of Agricultural Sciences, Harbin 150069, China; wzzxz20140105@126.com (Z.W.); zhupengyang2005@163.com (P.Z.); wuli198704@163.com (L.W.); shijianzhong@caas.cn (J.S.); liyanbing@caas.cn (Y.L.); 3 Department of Biology, College of Life Sciences, China Jiliang University, Hangzhou 310018, China

**Keywords:** ARNT, H5N1 influenza A virus, replication, interacting, PA

## Abstract

Increasing evidence suggests that the polymerase acidic (PA) protein of influenza A viruses plays an important role in viral replication and pathogenicity. However, information regarding the interaction(s) of host factors with PA is scarce. By using a yeast two-hybrid screen, we identified a novel host factor, aryl hydrocarbon receptor nuclear translocator (ARNT), that interacts with the PA protein of the H5N1 virus. The interaction between PA and human ARNT was confirmed by co-immunoprecipitation and immunofluorescence microscopy. Moreover, overexpression of ARNT downregulated the polymerase activity and inhibited virus propagation, whereas knockdown of ARNT significantly increased the polymerase activity and virus replication. Mechanistically, overexpression of ARNT resulted in the accumulation of PA protein in the nucleus and inhibited both the replication and transcription of the viral genome. Interaction domain mapping revealed that the bHLH/PAS domain of ARNT mainly interacted with the C-terminal domain of PA. Together, our results demonstrate that ARNT inhibits the replication of the H5N1 virus and could be a target for the development of therapeutic strategies against H5N1 influenza viruses.

## 1. Introduction

Influenza A virus is a major threat to humans and birds and has an enormous impact on the poultry industry and human public health. The H1, H2, and H3 subtypes of influenza virus have been successfully established in humans, and the H1N1 and H3N2 viruses are still circulating in humans worldwide [1,2]. Recent studies indicate that multiple avian influenza subtypes, such as H5, H6, H7, H9, and H10, not only affect the poultry industry but also cause sporadic or zoonotic human infections [3,4,5,6,7], with the highly pathogenic H5 and H7 viruses often causing severe disease [8]. The H7N9 viruses emerged in 2013 and caused more than 1560 human infections between 2013 and 2017 [7,9,10,11]; the H5 viruses have been identified worldwide in wild birds and poultry. As of 1 April 2022, H5 viruses have caused 938 human infections, including 487 deaths [8]. Therefore, the influenza viruses circulating in nature pose a substantial potential threat to public health.

Influenza virus replication is dependent on host cellular factors, and several cellular proteins and non-coding RNAs have been shown to facilitate or inhibit virus infection through different mechanisms [12,13,14,15,16,17,18,19,20,21,22,23,24,25]. The PA subunit is a multifunctional protein that mediates the transcription and replication of the influenza A virus [26,27]. It not only interacts with viral polymerase basic protein 2 (PB2) and polymerase basic protein 1 (PB1) to assemble RNA-dependent RNA polymerase (RdRp) but also interacts with host factors to regulate the replication and pathogenicity of influenza A viruses [28,29,30,31]. Several studies have identified hundreds of host factors that may interact with PA [32,33,34,35,36]; however, only a handful of PA-interacting host factors have been validated to date. Hsin et al. found that phosphorylation of RNA Pol II regulates the capping process of transcribed host RNAs by interacting with PA [37]. Huarte et al. reported that human CLE/C14orf166 protein (CLE) interacts with PA and is present in purified virus particles [38,39]. Interestingly, the expression of CLE increased after influenza virus infection, whereas considerable inhibition of the expression of other cellular proteins was observed in infected cells [40]. UBA52, another host factor essential for the replication of the influenza virus, interacts with all three PA gene-encoded proteins, PA, PA-N155, and PA-N182 [41]. Knockdown of UBA52 in chicken cells was shown to significantly inhibit the replication of the H5N1 virus and downregulate the host inflammation response. Other host factors, such as eEF1D, CHD6, and MCM, have also been shown to interact with PA and affect the replication of influenza viruses [14,42,43]. Given the importance of PA in the life cycle of influenza viruses, it is worthwhile investigating the role of host factors that interact with the PA protein during virus infection.

In our yeast two-hybrid screen, we identified a novel host factor, aryl hydrocarbon receptor nuclear translocator (ARNT), that interacts with the PA subunit of the H5N1 avian influenza virus. In this study, we explored the role of ARNT in the replication of the H5N1 virus in human cells. First, we confirmed the interaction between human ARNT and PA. Second, we mapped the domain that mediates the interaction, and finally, we assessed the biological role of ARNT in the life cycle of the H5N1 virus. Our study highlights the vital role of human ARNT in inhibiting the replication of the H5N1 virus.

## 2. Materials and Methods

### 2.1. Facility

Studies involving highly pathogenic H5N1 avian influenza virus were performed in a biosecurity level 3 facility approved by the Chinese Ministry of Agriculture at the Harbin Veterinary Research Institute (HVRI) of the Chinese Academy of Agricultural Sciences (CAAS).

### 2.2. Cells and Viruses

Human embryonic kidney cells (293T, Cat. No. CRL-3216) and human cervical cancer cells (HeLa, Cat. No. CCL-2) purchased from American Type Culture Collection (ATCC) were grown in Dulbecco’s modified Eagle’s medium (DMEM) supplemented with 10% fetal bovine serum (FBS, Sigma, St Louis, MO, USA) plus 1% penicillin–streptomycin antibiotics (Gibco, Waltham, MA, USA) at 37 °C in 5% CO_2_. Human lung epithelial A549 cells (Cat. No. CCL-185) purchased from ATCC were cultured in Ham’s F-12K medium supplemented with 10% FBS plus penicillin-streptomycin antibiotics (Gibco). The highly pathogenic H5N1 avian influenza A virus A/goose/Hubei/65/05 (GS/65) was isolated during the avian influenza outbreak in 2005 in China during the routine surveillance conducted by our laboratory. Viruses were propagated in 10-day-old specific-pathogen-free (SPF) embryonated chicken eggs and stored at −70 °C until use.

### 2.3. Plasmids Construction

Expression plasmids for the RdRp and nucleoprotein (NP) of GS/65, pcDNA3.1-65PB2, pcDNA3.1-65PB1, pcDNA3.1-65PA, and pcDNA3.1-65NP were constructed by inserting the indicated gene fragment into the pcDNA3.1 vector. To increase the protein expression, the PA gene inserted into the vectors was humanized and synthesized by the Genscript Company. The pCAGGS-PA-Flag was a pCAGGS plasmid with the humanized PA gene with a Flag tag at its C-terminus; the human ARNT gene was PCR-amplified from the cDNA of HeLa cells and then cloned into the pCAGGS vector with or without a c-Myc tag at the C-terminus to obtain pCAGG-ARNT-c-Myc and pCAGGS-ARNT, respectively. ARNT mutants were constructed by inserting different length ARNT fragments with a c-Myc tag at the C-terminus into the pCAGGS vector. PA mutants were generated by inserting various lengths of PA fragments into pCAGGS. To generate plasmids expressing fluorescent proteins, PA and ARNT fused with eGFP and DsRed were inserted into the pEGFP-N1 or pDsRed-Monomer-N1 vector (Clonetech, Mountain View, CA, USA), generating peGFP-PA and pDsRed-ARNT, respectively. All plasmids were sequenced to confirm the absence of unwanted mutations. The primers are available upon request.

### 2.4. Co-Immunoprecipitation and Western Blot Analysis

293T cells were transfected with the indicated plasmids using Lipofectamine 2000 reagent (Invitrogen, Waltham, MA, USA). After 48 h, the cells were washed twice with cold phosphate-buffered saline (PBS) and then lysed in IP buffer (50 mM Tris-HCl pH 7.4, 150 mM NaCl, 1 mM EDTA, 1% NP-40, and 5% glycerol) containing a complete protease inhibitor cocktail (Roche, Basel, Switzerland) on ice for 30 min with periodic mixing by vortexing. Cell debris was removed by centrifugation at 12,000× *g* for 10 min at 4 °C, and then cell lysates were precleared with 20 μL of Dynabeads Protein G (Life Technologies, Carlsbad, CA, USA). After treatment, the lysates were incubated with the indicated antibody at 4 °C overnight, and then Dynabeads Protein G was added, and the lysates were gently rotated for 6 h at 4 °C. The beads were then washed four times with IP buffer on a magnetic rack. The bound proteins were separated by sulfate-polyacrylamide gel electrophoresis (SDS-PAGE), followed by Western blotting with the indicated antibodies. The nitrocellulose membranes were scanned on an Odyssey Infrared Imaging System (LiCor, Lincoln, NE, USA).

### 2.5. Antibodies

Antibodies were obtained from the following sources: PA antibody (customized from Genscript), NP antibody (Immune Technology, New York, NY, USA, IT-003-023), and β-actin (Santa Cruz, sc-69879) antibodies were purchased from Santa Cruz; Mouse anti-ARNT monoclonal antibody (Santa Cruz, sc-55526); Rabbit anti-ARNT monoclonal antibody (Cell Signaling Technology, 3414s, Danvers, MA, USA). Alexa Fluor 680 donkey anti-rabbit IgG and Alexa Fluor 680 donkey anti-mouse IgG antibodies were purchased from Invitrogen.

### 2.6. Immunofluorescence

293T cells were grown on glass-bottom dishes and were transfected with the indicated plasmid(s). At 24 h post-transfection (p.t.), the cells were fixed with 4% paraformaldehyde in PBS for 20 min at room temperature and permeabilized with 0.5% Triton X-100 in PBS for 20 min. After being blocked with 5% bovine serum albumin (BSA) in PBS for 1 h, the cells were incubated with rabbit antisera against PA (Santa Cruz) or a mouse monoclonal antibody against ARNT (Santa Cruz) at 4 °C overnight. After being washed three times with PBS, the cells were incubated for 1 h with the Alexa Fluor™ 488 donkey anti-mouse IgG (H + L) highly cross-adsorbed secondary antibody and Alexa Fluor™ 594 donkey anti-rabbit donkey anti-rabbit IgG (H + L) highly cross-adsorbed secondary antibody (Invitrogen). After incubation for 1 h, the cells were washed three times with PBS and stained with 4′,6-diamidino-2-phenylindole (DAPI) for 10 min. Cells were observed by confocal laser scanning microscopy (Leica, Wetzlar, Germany).

### 2.7. Virus Infection

To infect cells overexpressing ARNT, 293T cells were transfected with the pCAGGS-hANRT plasmid and were then infected with GS/65 at a multiplicity of infection (MOI) of 0.001 at 24 h p.t. for 1 h. After washing the cells three times with opti-MEM, the cells were cultured at 37 °C. Supernatants were collected at the indicated time points and titrated in 10-day-old embryonated eggs. To infect ARNT knocked down cells, first, Hela cells were transfected with ARNT-specific siRNA (synthesized from Invitrogen [44]) at a concentration of 100 nM by using Lipofectamine RNAiMax reagent (Invitrogen). Non-targeting siRNA (siScr) was used as a control. After knockdown was confirmed by qRT-PCR and Western blotting, HeLa cells transfected with siRNA for 48 h, were washed three times with Opti-MEM, and then infected with GS/65 at an MOI of 0.1 for 1 h. After washing three times with Opti-MEM, the cells were cultured at 37 °C. The supernatants were collected at the indicated time points, and the virus titers were determined in 10-day-old embryonated eggs. To detect the interaction between viral PA and ARNT, HeLa cells were infected with GS/65 at an MOI of 2 for 24 h, and the indicated proteins were probed as described above.

### 2.8. Minigenome Replicon Assay

The effect of ARNT on the polymerase activity of GS/65 was analyzed in both ARNT overexpressing cells and ARNT knocked down cells by using a minigenome replicon assay. To evaluate the effect of ARNT overexpression on the polymerase activity, 293T cells were co-transfected with a plasmid expressing ARNT (0.8 μg), four plasmids encoding the polymerase subunits (PA, PB1, and PB2), and NP protein (0.4 μg, each plasmid), a plasmid expressing luciferase from a virus-like RNA encoding the *Firefly* luciferase (pPolI-T-Luc, 0.4 μg), and an internal control vector expressing the *Renilla* luciferase protein (pTK-RL, 0.4 μg). At 36 h p.t., luciferase activities were determined by using the Dual-Glo luciferase assay system (Promega) as previously described [28]. To evaluate the effect of ANRT knockdown on the polymerase activity, HeLa cells were transfected with ARNT-specific siRNA. Forty-eight hours after transfection, HeLa cells were transfected with plasmids expressing PB2, PB1, PA, NP, pPolI-T-Luc, and pTK-RL. The polymerase activity was analyzed as described above at 48 h p.t. The assay was standardized against the *Renilla* luciferase activity. All experiments were performed in triplicate.

### 2.9. Quantitative Reverse Transcription-PCR Assay (qRT-PCR)

To detect the efficiency of ARNT knockdown, the treated HeLa cells were washed three times with PBS, and then the RNA was extracted by using Trizol reagent (Invitrogen). A portion of the total RNA was reverse transcribed by using the PrimeScript RT reagent kit and detected by using the SYBR Premix ExTaq II kit (Takara, Kusatsu, Japan). To synthesize the cDNA of vRNA, mRNA, and cRNA of the NP gene, a vRNA-specific primer complementary to the 3′ end of vRNA, oligo(dT) primer, and a cRNA-specific primer, respectively, were used. Each sample was normalized based on the amount of β-actin. For quantification of vRNA, cRNA, and mRNA, the SYBR green-based real-time PCR method (Takara) was used. Primers used in this assay were as follows: NP-Forward (5′-GAGACGGAAAATGGGTGAGG-3′) and NP-Reverse (5′-ATCAGGTGGGTAAGACCAGCA-3′); β-actin-Forward (5′-TCGTGCGTGACATTAAGGAGA-3′) and β-actin–Reverse (5′-GGATGTCCACGTCACACTTCA-3′). All real-time PCR experiments were performed by using the LightCycler 480 system (Roche).

### 2.10. Statistical Analyses

The significance of the difference between groups was analyzed with the Student’s *t*-test. A *p*-value of less than 0.05 was considered statistically significant.

## 3. Results

### 3.1. ARNT Interacts with the PA Protein of Influenza Virus

**Figure 1:**
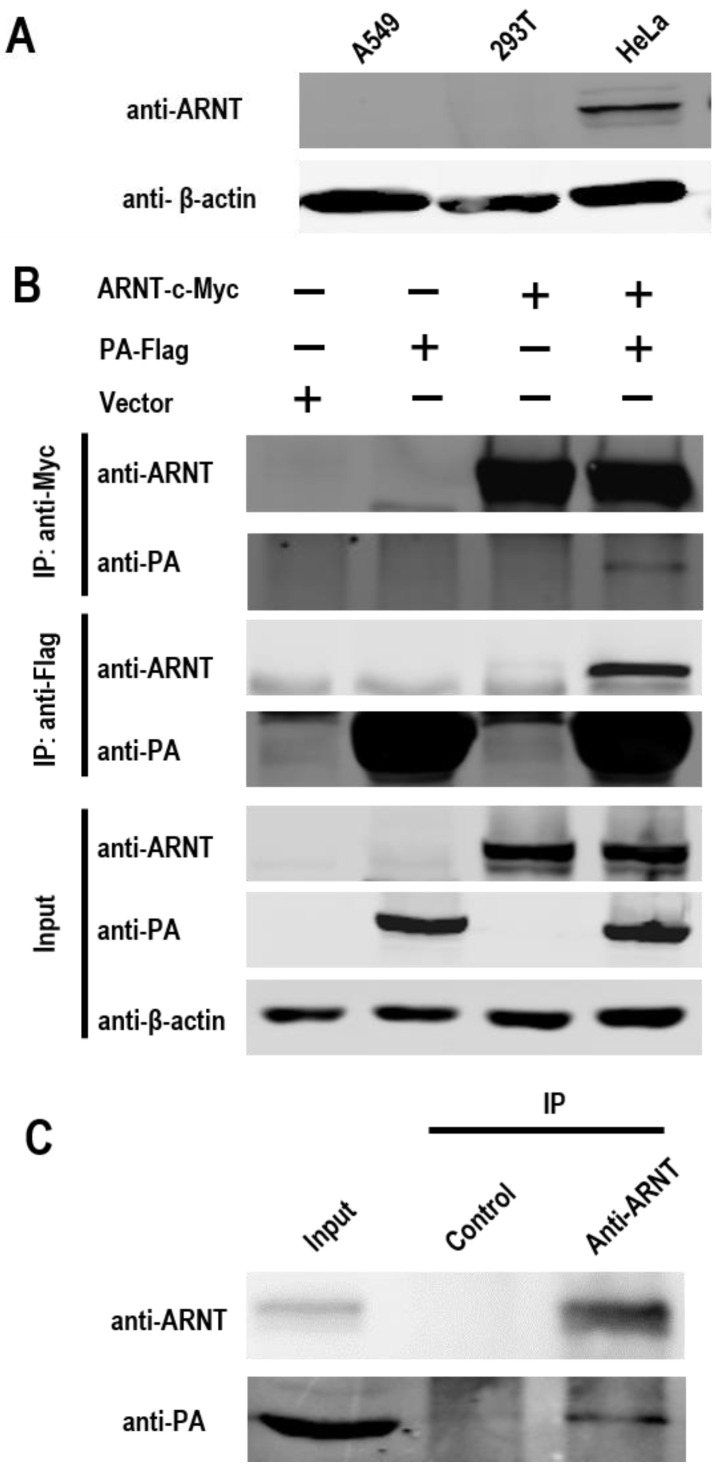
The interaction between ARNT and PA was analyzed by using co-immunoprecipitation assay. (**A**) The endogenous expression of ARNT in A549, 293T, and HeLa cells. (**B**) The interaction between PA and ARNT in 293T cells. 293T cells were co-transfected with the pCAGGS-PA-Flag and pCAGGS-ARNT-c-Myc plasmids. The cell extracts were subjected to co-immunoprecipitation assays by using either anti-Flag or anti-c-Myc antibodies to capture the immune complex. The bound proteins were then analyzed reciprocally by Western blotting with an anti-PA antibody to detect anti-c-Myc immunoprecipitates and an anti-ARNT antibody to detect anti-Flag immunoprecipitates. (**C**) The interaction between viral PA and endogenous ARNT in HeLa cells. HeLa cells were infected with GS/65 at an MOI of 2. At 24 h p.i., the cell extracts were immunoprecipitated with a rabbit anti-ARNT antibody or anti-control IgG antibody, followed by Western blotting with an anti-ARNT antibody and an anti-PA antibody.

Through our yeast two-hybrid screen (not published data), the novel host factor ARNT was identified as an interacting partner for the PA protein of A/goose/Hubei/65/2005 (H5N1, GS/65). Here, we further investigated the interaction between PA and human ARNT in cells. To select a suitable cell line for further studies, the endogenous ARNT of three human cell lines, 293T, A549, and HeLa cells, was evaluated. The data indicated that ARNT was highly expressed in HeLa cells, whereas no or low levels of ARNT were expressed in 293T and A549 cells, respectively (Figure 1A).

To investigate whether PA physically interacts with ARNT in human cells, we performed a co-immunoprecipitation assay in 293T cells, which are usually with a high transfection efficacy and are with no detectable endogenous ARNT expression. 293T cells were co-transfected with a plasmid expressing the PA protein fused with a Flag tag (pCAGGS-PA-Flag) and a plasmid expressing ARNT fused with a Myc tag (pCAGGS-ARNT-c-Myc). Forty-eight hours p.t., the cell lysates were immunoprecipitated with either anti-Flag or anti-c-Myc antibodies, then the PA and ARNT were detected by Western blotting. We found that ARNT co-precipitated with PA and vice versa (Figure 1B). Next, the interaction between endogenous ARNT and the viral PA of GS/65 was examined by co-immunoprecipitation in HeLa cells with a high expression of endogenous ARNT. HeLa cells were infected with GS/65 at an MOI of 2, and then the cell lysates were precipitated with an ARNT antibody, followed by detection with an anti-PA antibody. As expected, the PA protein was successfully precipitated by ARNT (Figure 1C). These data demonstrate that PA physically interacts with ARNT.

### 3.2. The bHLH/PAS Domain of ARNT Interacts with PA

**Figure 2:**
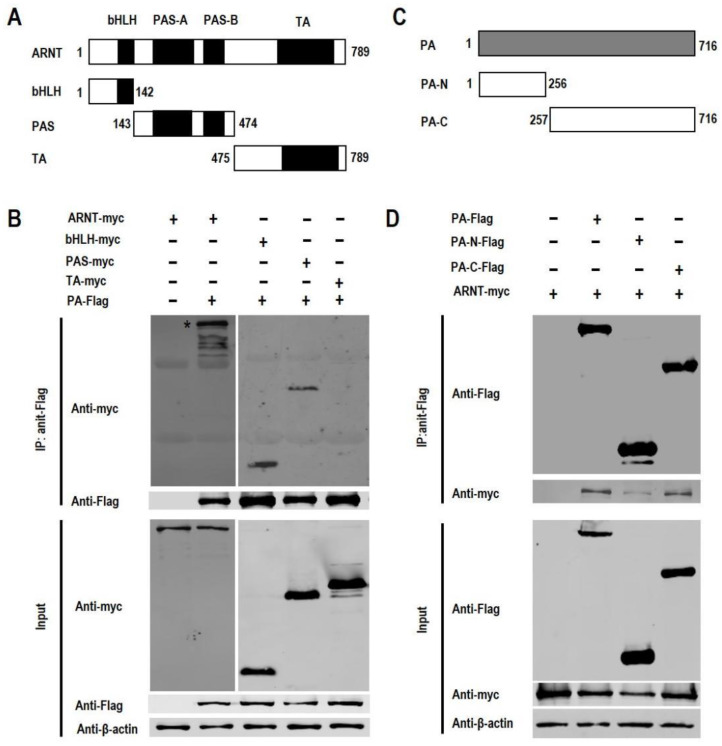
Interaction between subdomains or domains of ARNT and PA in 293T cells. (**A**) Schematic diagram of the full-length ARNT and ARNT mutants tested in this study. (**B**) Co-immunoprecipitation analysis of the interaction between PA and ARNT mutants. 293T cells were co-transfected with pCAGGS-PA-Flag and different c-Myc-tagged ARNT mutants. At 48 h p.t., cell lysates were immunoprecipitated with an anti-Flag antibody followed by Western blotting with anti-c-Myc and anti-Flag antibodies. (**C**) Schematic diagram of the full-length PA and PA mutants. (**D**) Co-immunoprecipitation analysis of the interaction between PA mutants and ARNT. 293T cells were co-transfected with pCAGGS-ARNT-c-Myc and different Flag-tagged PA mutants. At 48 h p.t., cell lysates were immunoprecipitated with an anti-Flag antibody followed by Western blotting analysis with anti-c-Myc and anti-Flag antibodies. *, the target protein.

ARNT comprises a basic helix-loop-helix domain (bHLH), a PER-ARNT-SIM (PAS) domain (including PAS A and PAS B), and a transcription activation (TA) domain (Figure 2A). To identify which domain(s) of ARNT interact(s) with PA, the three domains of ARNT were expressed individually, and their interaction with PA was analyzed by use of the co-immunoprecipitation assay in 293T cells. We found that both the bHLH domain and the PAS domain interact with PA, but no interaction between the TA domain and PA was detectable (Figure 2B).

To investigate which domain(s) of PA interact(s) with ARNT, the PA protein was split into the N-terminal domain (PA-N) and C-terminal domain (PA-C) (Figure 2C), and the interaction between ARNT and the two domains of PA was analyzed by co-immunoprecipitation in 293T cells. We found that ARNT was precipitated with both PA-N and PA-C, but the amount of ARNT that was precipitated by PA-C was clearly higher than that precipitated by PA-N (Figure 2D). These data indicate that ARNT interacts with both PA-N and PA-C, but its affinity for these two domains of PA may be different.

### 3.3. Overexpression of ARNT Decreases the Polymerase Activity and Inhibits the Replication of H5N1 Influenza Virus

**Figure 3:**
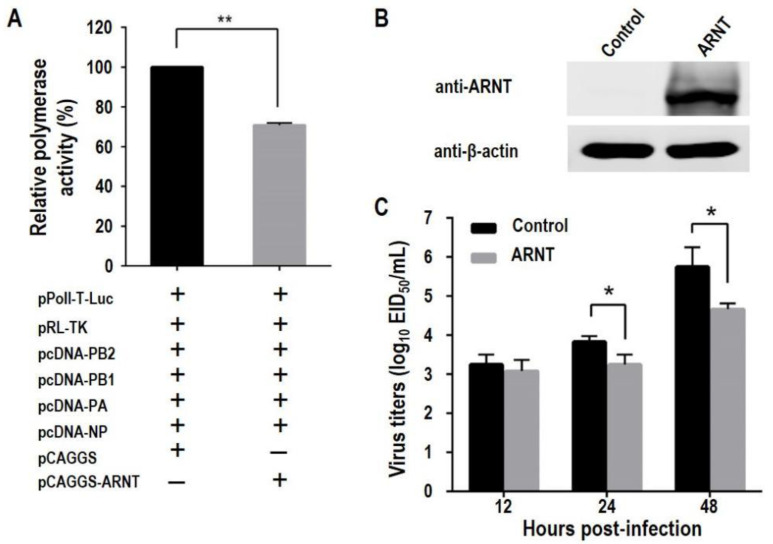
The polymerase activity and replication of influenza virus in 293T cells overexpressing ARNT. (**A**) The effect of ARNT on the polymerase activity of GS/65. 293T cells were transfected with two plasmids expressing *Firefly* and *Renilla* luciferases and the other indicated plasmids. At 36 h p.t., the cell lysates were subjected to the luciferase assay described in the Materials and Methods. The overexpression of ARNT inhibits the replication of GS/65 (**B**,**C**). 293T cells were transfected with pCAGGS-ARNT and pCAGGS empty vector, and the overexpression of ARNT in 293T cells was confirmed by Western blotting (**B**). At 24 h p.t., the cells were infected with GS/65 at an MOI of 0.001. The supernatants were collected at the indicated timepoint. Virus titers in the supernatants were determined in embryonated eggs (**C**). * *p* < 0.05; ** *p* < 0.01.

To investigate whether overexpression of ARNT affects the polymerase activity of influenza virus, 293T cells were transfected with four plasmids expressing PB2, PB1, PA, and NP of GS/65, two plasmids expressing *Firefly* and *Renilla* luciferases, together with or without ARNT expressing plasmid pCAGGS-ARNT. We found that the polymerase activity in cells co-transfected with ARNT was only 70.8% of that in the cells transfected without ARNT (Figure 3A).

Since the polymerase activity is directly related to the replication of the influenza virus, viruses with higher polymerase activity have higher replication titers in cells [16,18,45,46,47]; we evaluated the effect of ARNT on the replication of GS/65. Control 293T cells and 293T cells overexpressing ARNT, which was confirmed by Western blotting (Figure 3B), were infected with GS/65 at an MOI of 0.001, and the cell supernatants were collected at different time points post-infection (p.i.) and titrated in eggs. We found that the viral titers in the control 293T cells were significantly higher than those in the 293T cells overexpressing ARNT at 24 h and 48 h p.i., although the viral titers were comparable at 12 h p.i. (Figure 3C). These results indicate that overexpression of ARNT decreases the polymerase activity and inhibits the replication of influenza virus in cells.

### 3.4. Knockdown of ARNT Increases the Polymerase Activity and Promotes the Replication of H5N1 Influenza Virus

**Figure 4:**
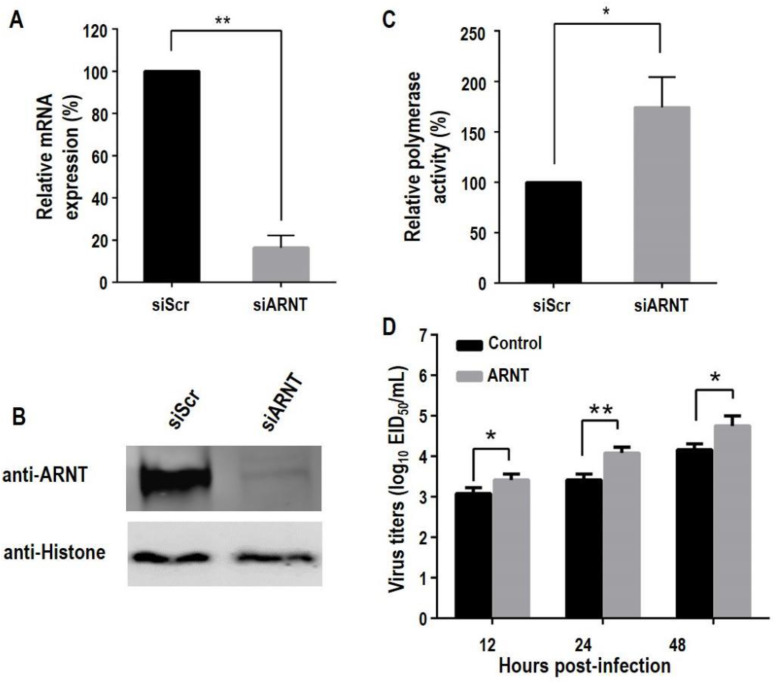
The polymerase activity and replication of influenza virus in HeLa cells with ARNT knockdown. Knockdown of ARNT in HeLa cells. HeLa cells were transfected with ARNT-specific siRNA (siARNT) and non-targeting siRNA (siScr) at a concentration of 100 nM. At 48 h p.t., the total RNA and nuclear proteins were extracted for qRT-PCR (**A**) and Western blotting (**B**) to evaluate the ARNT knockdown efficiency. (**C**) The effect of ARNT knockdown on the polymerase activity of H5N1 influenza A virus. Four RNP-expressing plasmids along with the *Firefly* and *Renilla* luciferase-expressing plasmids (pPolI-T-Luc and pRL-TK) were co-transfected into HeLa cells pretreated with either siARNT or siScr. (**D**) The effect of ARNT knockdown on the replication of H5N1 influenza A virus. HeLa cells, transfected with siARNT or siScr for 48 h, were infected with GS/65 at an MOI of 0.1. Cell supernatants were collected at 12, 24, and 48 h p.i. and titrated in eggs. * *p* < 0.05; ** *p* < 0.01.

To confirm the role of ARNT knockdown in the replication of influenza virus, we evaluated the polymerase activity and growth of GS/65 in ARNT knockdown HeLa cells, which are with a high expression of endogenous ARNT. HeLa cells were transfected with ARNT-specific siRNA (siARNT), and knockdown efficiency of ARNT was confirmed by quantitative RT-PCR (qRT-PCR) (Figure 4A) and Western blotting (Figure 4B). We found that the polymerase activity in the ARNT knockeddown cells was 174.2% of that in the control cells (Figure 4C). To investigate the effect of ARNT on virus replication, control HeLa cells and siARNT-transfected HeLa cells were infected with GS/65 at an MOI of 0.1, and the supernatants were collected at different time points p.i. and titrated in eggs. We found that the viral titers in the siARNT-transfected HeLa cells were significantly higher than those in control cells at all time points (Figure 4D). These results indicate that knockdown of ARNT increases the polymerase activity and promotes the replication of the H5N1 influenza virus.

### 3.5. ARNT Inhibits Viral Genome Transcription and Replication

**Figure 5:**
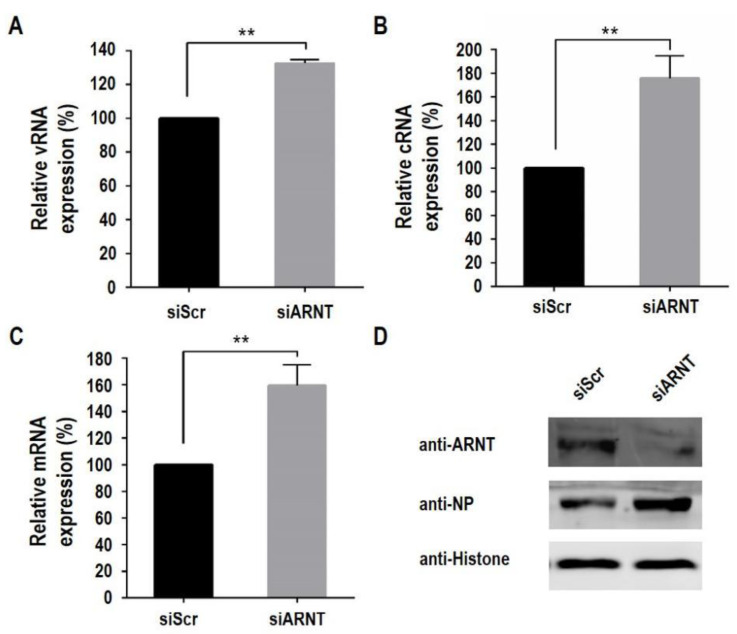
The effect of ARNT on viral genome replication and transcription. HeLa cells treated with ARNT-specific siRNA (siARNT) and non-targeting siRNA (siScr) were infected with GS/65 virus at an MOI of 2. At 8 h p.i., the RNA was extracted, and then the expression levels of vRNA (**A**), cRNA (**B**), and mRNA (**C**) of the NP gene were determined by using qRT-PCR. Nuclear proteins were extracted for Western blot analysis (**D**). ** *p* < 0.01.

Since the polymerase activity and replication of GS/65 were found to be negatively regulated by ARNT, and the polymerase is involved in the synthesis of vRNA, cRNA, and mRNA, we next investigated which type of RNA production is regulated by ARNT. Control HeLa cells and siARNT-transfected HeLa cells were infected with GS/65 at an MOI of 1, and the vRNA, cRNA, and mRNA of the NP gene were analyzed by qRT-PCR. We found that the level of all three types of RNA was significantly higher in the siARNT-transfected HeLa cells than in the control HeLa cells (Figure 5A–C) and that the expression of viral NP in the siARNT-transfected HeLa cells was also notably higher than that in the control HeLa cells (Figure 5D). These results indicate that ARNT inhibits both viral RNA replication and transcription.

### 3.6. ARNT Results in the Nuclear Accumulation of the PA Protein

**Figure 6:**
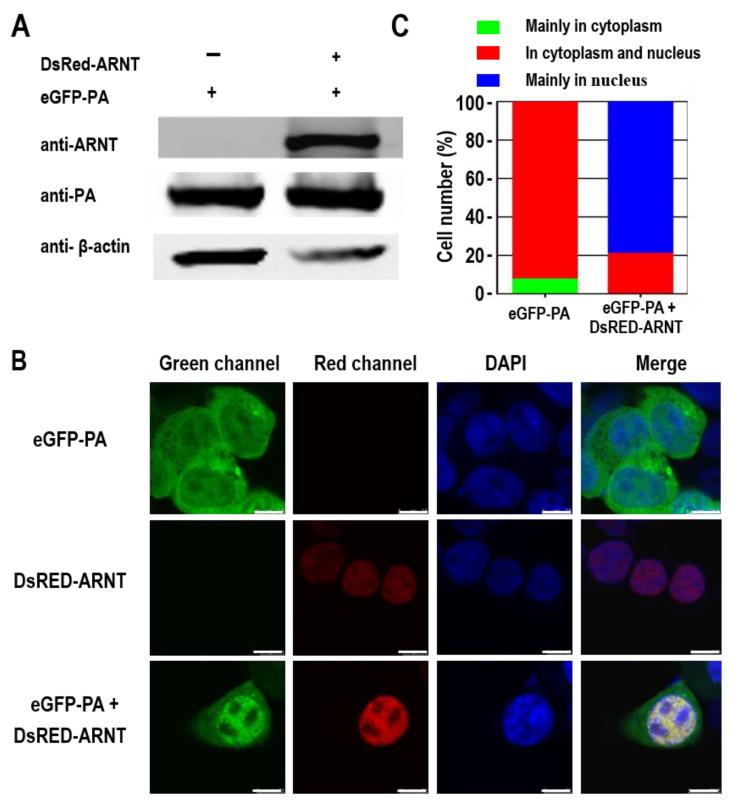
The effect of ARNT on the distribution of PA protein in 293T cells. (**A**) Confirmation of the expression of eGFP-PA and DsRed-ARNT in 293T cells by Western blotting. (**B**) The distribution of eGFP-PA and DsRed-ARNT in cells. 293T cells were transfected with peGFP-PA and pDsRED-ARNT individually or in combination. At 24 h p.t., the cells were fixed, and the nuclei were stained by DAPI. The cells were then scanned using a confocal microscope. Typical images are presented. (**C**) Statistical analysis of the intracellular localization patterns of PA in 293T cells. Based on the confocal microscopy data in panel A, the localization of PA was categorized into three types: clear nuclear localization (blue), simultaneous localization in the cytoplasm and nucleus (red), and predominantly cytoplasmic localization (green). The localization of PA was analyzed in more than 50 cells. The scale bar indicates 0–7.5 μm in length.

ARNT has been reported to bind to ligand-bound aryl hydrocarbon receptor (AhR) and aid in its nuclear transport [48]. Since PA functions in the nucleus during viral vRNA and mRNA synthesis, we investigated whether its interaction with ARNT affects its nuclear transport. To this end, 293T cells were transfected with peGFP-PA (PA fused to the enhanced green fluorescent protein) and pDsRed-ARNT (ARNT fused to Discosoma red fluorescent protein) alone or together, and the localization of PA and ARNT in the transfected cells was analyzed by using confocal microscopy. The expression of ARNT and PA in the transfected cells was confirmed by Western blotting (Figure 6A). We found that the red fluorescence was localized in the nucleus of cells transfected with pDsRed-ARNT and that green fluorescence was detected in both the cytoplasm and nucleus of cells transfected with peGFP-PA (Figure 6B). However, the green fluorescence was mainly detected in the nucleus of cells co-transfected with peGFP-PA and pDsRed-ARNT (Figure 6B), and PA was detected in both the cytoplasm and nucleus (Figure 6B). Quantitative analysis indicated that for 7.7% of the cells, PA mainly accumulated in the cytoplasm, and for the remaining 92.3% of the cells, PA was located in both the nucleus and cytoplasm when the cells were transfected with peGFP-PA. In contrast, when the cells were co-transfected with peGFP-PA and pDsRed-ARNT, 21.1% showed PA distributed in both the nucleus and cytoplasm, and for the other 78.9% of the cells, PA accumulated in the nucleus (Figure 6C). These results indicate that ARNT results in the nuclear accumulation of the PA protein.

## 4. Discussion

The influenza virus has to rely on host factors to complete its life cycle. Therefore, the interactions between viral proteins and host proteins are critical to the replication of influenza viruses. There is increasing evidence that the PA of influenza A virus affects the virulence and transmission of influenza viruses [18,21,30,36,49,50,51], suggesting that PA also contributes to the host adaptation. In this study, we identified and proved that the cellular protein ARNT is a host factor that interacts with the PA of the influenza virus. We also found that ARNT facilitates the nuclear accumulation of PA. Overexpression of ARNT inhibits the transcription and replication of the viral genome and reduces viral replication in human cells. Our data show that ARNT is an intrinsic host factor that negatively regulates the replication of the H5N1 influenza virus.

ARNT, also known as hypoxia-inducible factor (HIF)-1β, is a member of the basic bHLH/PAS family of transcription factors. It acts as a binding partner for AhR as well as the HIF-1α subunit and plays an important role in the adaptive response to (micro) environmental stressors such as dioxin exposure and hypoxia [48]. Studies of bHLH/PAS proteins indicate that these proteins mediate the transcriptional activation of many genes associated with pathological stressors, such as inflammation, infectious microorganisms, and cancer [48,52,53]. Contrary to its binding partner, HIF-1α, whose role in virus infection has been extensively investigated, the antiviral activity of ARNT has not been well studied. HIF-1α not only regulates the replication of the influenza virus [54,55,56], but also that of other viruses, such as a respiratory syncytial virus (RSV) [57], hepatitis B and C viruses (HBV and HCV) [58], and severe acute respiratory syndrome coronavirus 2 viruses (SARS-CoV-2) [59]. Choi et al. reported that knockdown of HIF-1β by siRNA did not affect the expression of HIF-1α [44]; therefore, the inhibitory effect of HIF-1β was not through affecting the expression of HIF-1α. In this study, we provide direct evidence that ARNT is a novel intrinsic antiviral factor that restricts the replication of the H5N1 virus. The effect of ARNT on human influenza viruses and other viruses remains to be examined.

Polymerase activity plays a critical role in the pathogenicity, transmission, and host adaptation of the influenza virus [16,18,21,45,46,47,60]. Moreover, several studies have shown that PA plays an essential role in the activity of RdRp and the cross-species transmission of the influenza A virus. Mehle et al. found that PA T552S (552S possessed by the pandemic 2009 H1N1 virus) increases viral replication and contributes to the cross-species transmission of an avian influenza virus [30]. Zhang et al. reported that PA from the pandemic 2009 H1N1 virus promotes the polymerase activity and transmission of H5 avian influenza virus in guinea pigs [50]. Liang et al. reported that the low polymerase activity of avian influenza virus in mammals was caused by PA protein [18]. In this study, we demonstrated that ARNT restricts virus replication by inhibiting polymerase activity. Several host factors have been found to inhibit polymerase activity via different mechanisms. Chen et al. reported that the host factor HDAC6 suppresses the polymerase activity of the H1N1 virus via the deacetylation of PA [61], resulting in decreased virus replication. Gao et al. reported that the host factor NCL interacts with PA and inhibits the polymerase activity and replication of the H5N1 virus [62]. Like ARNT, NCL was found to interact with PA mainly in the nucleus. Hsu et al. showed that the host factor HAX1 downregulates the polymerase activity of H1N1 by interacting with the nuclear localization signal in the N-terminal domain of PA [63]. Further studies revealed that HAX1 inhibits the nuclear accumulation of PA. In this study, the bHLH domain of ARNT strongly interacts with PA. Analysis of duck, chicken, and human ARNT indicated that the bHLH domain of ARNT is highly conserved (~91% similarity in amino acid), which may indicate that avian ARNT may exhibit an inhibitory effect on the H5N1 virus as well. Our data indicate that ARNT strongly interacts with the PA-C domain and leads to the nuclear accumulation of PA. We speculate that ARNT may restrict the replication of the influenza virus through two possible mechanisms: (1) Impeding the formation of RdRp. The PA-C domain is thought to be involved in the interaction with PB1 [27]; therefore, the formation of RdRp may be affected by the interaction between PB1 and ARNT, which may be caused by the steric hindrance of ARNT. (2) Impeding the dimerization of HIF-1α/ARNT or AhR/ARNT. Previous studies indicate that the bHLH/PAS domain of ARNT is involved in the dimerization of HIF-1α/ARNT or AhR/ARNT; as a result, the transcriptional activation of many genes associated with pathological stressors may be affected [48,52,53]. Additional studies are needed to explore the molecular mechanisms of ARNT-mediated inhibition of viral replication.

In summary, here, we investigated the role of ARNT in the life cycle of the influenza virus. We demonstrated that ANRT negatively regulates the polymerase activity and replication of the H5N1 influenza virus. ARNT mainly interacts with the C-terminal domain of PA and leads to the nuclear accumulation of PA. Our findings highlight a new role for ARNT in inhibiting the replication of the H5N1 influenza virus and open the door to the development of ARNT-derived peptides with therapeutic potential.

## 5. Conclusions

Increasing evidence suggests that the PA of the influenza A viruses plays an important role in viral replication and pathogenicity. However, information regarding the interaction(s) of host factors with PA is limited. In this study, we extend this knowledge by identifying a novel host factor, ARNT, that interacts with the PA protein of the H5N1 virus. The ARNT mainly interacts with the C-terminal domain of PA. Mechanistically, overexpression of ARNT resulted in the accumulation of PA protein in the nucleus and inhibited both the polymerase activity and viral replication. Knockdown of ARNT promotes the replication and transcription of viral genome and virus growth. Our results suggested that ARNT is a novel restriction factor of the H5N1 influenza virus and could be a potential target for developing therapeutic strategies against the H5N1 influenza virus.
